# Effects of Mindful Eating and YogaDance among Overweight and Obese Women: An Exploratory Randomized Controlled Trial

**DOI:** 10.3390/nu15071646

**Published:** 2023-03-28

**Authors:** Sofie Hauerberg Henninger, Anna Yde Fibieger, Faidon Magkos, Christian Ritz

**Affiliations:** 1 Department of Nutrition, Exercise and Sports, University of Copenhagen, DK-1958 Frederiksberg, Denmark; 2 Department of National Institute of Public Health, University of Southern Denmark, DK-1455 Copenhagen, Denmark

**Keywords:** eating behavior, fat mass, mindful eating, mental health, quality of life, yoga, dance

## Abstract

Many current treatment options for managing overweight and obesity consist of rather strict diet and exercise regimes that are difficult to implement as a lifelong routine. Therefore, alternative initiatives such as mindful eating and pleasure-oriented physical activity with more focus on implementation and enjoyment are needed to reverse the obesity epidemic. Mindful eating is an approach focusing on inner hunger and satiety signals. YogaDance is a novel exercise approach combining elements of yoga and dance. This study was a randomized controlled trial investigating the individual and combined effects of mindful eating and YogaDance. Participants were healthy, inactive women with overweight or obesity (body mass index ≥ 25 kg/m^2^ and/or waist circumference ≥ 80 cm) who were randomized to one of four groups for 8 weeks: mindful eating alone, YogaDance alone, the combination of mindful eating and YogaDance, or control. Fat mass was the primary outcome and secondary outcomes included body weight, waist circumference, and other physiological, behavioral, and quality-of-life outcomes. Sixty-one women were included in the study and randomized to mindful eating and YogaDance combined, YogaDance, mindful eating, or control. Fat mass was reduced by 1.3 kg (95% CI [−10.0, 7.3] kg; *p* = 0.77), 3.0 kg (95% CI [−11.1, 5.1] kg; *p* = 0.48), and 1.8 kg (95% CI [−10.1, 6.6] kg; *p* = 0.69) for the mindful eating, YogaDance, and combined mindful eating and YogaDance interventions, respectively, compared to the control, with corresponding effect sizes of 0.15, 0.34, and 0.21. In complete-case analyses, fat percent and waist circumference were reduced whereas mental quality of life and eating behavior were improved for mindful eating and mindful eating and YogaDance combined compared to the control. In conclusion, the study found modest benefits of an 8-week combination of mindful eating and YogaDance, corroborating findings in previous studies on mindful eating, yoga, and dance. However, the study had several limitations that should be taken into consideration, including low power due to a large drop-out as well as low to moderate training load and compliance. The trial was retrospectively registered (ISRCTN87234794).

## 1. Introduction

The cornerstones for management of overweight and obesity are lifestyle modifications, which often involve calorie-restricted diets and/or demanding exercise programs. For instance, the benefits of regular exercise on the general health of adults are well-established [1,2]. However, such programs are often shortsighted and not implementable as a lifelong routine [3]. Mental health may be negatively affected due to failure to adhere to the program requirements and experiencing unsuccessful weight loss [4]. For women the risk of disordered eating has also been pointed out [5]. Therefore, novel weight loss interventions with a greater focus on long-term implementation and enjoyment may offer attractive alternatives for the management of overweight and obesity in women.

Two such approaches are mindful eating and YogaDance. Mindful eating has recently attracted much attention in nutrition research [6,7]. In short, mindful eating aims at aligning eating habits with inner hunger and satiety signals rather than counting calories [8,9]. The eating situation is centered around utilizing the senses, being calm and relaxed and enjoying the food to the fullest, disrupting the tendency to overeat, and thereby reducing energy intake [10]. Mindful eating has been shown to be as effective as conventional weight loss interventions [11]. Moreover, mindful eating has been shown to positively affect eating behavior [12].

YogaDance is a novel training concept developed by the authors S.H.H. and A.Y.F. YogaDance combines elements of yoga and dance that complement each other, exploiting the fact that both yoga and dance have been shown to improve body composition and related outcomes. Yoga is a popular trend in the global health and fitness industry [13]. Specifically, yoga has been shown to reduce weight, waist circumference, and body mass index (BMI) [14,15]. Likewise, there is some evidence supporting the claim that dance improves mental health and quality of life [16,17]. The combination of these two approaches incorporates both strength and endurance exercises as well as mindfulness and relaxation, and it adds an enjoyable, fun, and playful aspect into training.

The aim of this study was to explore the effects of combining mindful eating and YogaDance in overweight and obese women. The hypothesis was that the combination would lead to a reduction in total body fat mass (compared to a control group). Beneficial effects for other physiological outcomes and improved quality of life and eating habits were also anticipated.

## 2. Materials and Methods

### 2.1. Trial Design

This study was an exploratory open-label, parallel-arm, randomized controlled superiority trial, where participants were allocated by block randomization to one of three intervention groups (mindful eating alone, YogaDance alone, or mindful eating and YogaDance combined; see below for more details) or the control group in a 1:1:1:1 ratio. This trial design enabled assessment of the two approaches—mindful eating and YogaDance separately as well as jointly. The trial was retrospectively registered in February 2023, in the ISRCTN registry (ISRCTN87234794).

### 2.2. Sample Size Calculation

Heydari and colleagues found a reduction in fat mass of 2.1 kg between high-intensity intermittent exercise and no exercise groups; the study also provided an SD of 1.9 kg [18]. Therefore, in the present study, an effect size of 2.1/1.9 = 1.1 was assumed to be of a practically relevant size. Assuming a significance level of 0.05 and a power of 80%, there were 13 participants per group, corresponding to a total of 52 participants. Furthermore, assuming a 15% dropout resulted in a final sample size of 60.

### 2.3. Participants 

Participants were recruited via social media on Facebook and Instagram as well as on the Danish websites www.forsøgsperson.dk and www.nexs.ku.dk (advertised from November 2021 to January 2022). Flyers and posters in the area of Copenhagen were also used for recruitment. To be included in the study, participants had to be women between 18 and 65 years of age, with a body mass index ≥ 25 kg/m^2^ and/or a waist circumference ≥ 80 cm. Furthermore, participants should be healthy and have an inactive lifestyle defined as <2.5 h of light physical activity per week or <1 h of moderate-to-hard physical activity per week. Participants were not eligible if they were pregnant, breast-feeding, or planned to become pregnant within the study period. Other exclusion criteria were any self-reported severe disease (including cancer, CVD, type 1 or 2 diabetes, osteoporosis), a self-reported eating disorder (e.g., anorexia, bulimia, orthorexia, binge eating disorder), following a very restrictive diet (not eating several food items), and participation in other studies.

### 2.4. Intervention

#### 2.4.1. Intervention Groups

The intervention period was 8 weeks. The YogaDance intervention group followed three weekly YogaDance classes (in total. 24 classes), whereas the mindful eating group attended a workshop every second week (in total four workshops). The combined mindful eating and YogaDance intervention group followed both three weekly YogaDance classes and a mindful eating workshop every second week. The control group did not participate in the YogaDance classes nor the mindful eating workshops. During the intervention period, volunteers were told to avoid other strenuous physical activities.

To increase compliance and willingness to participate in the study, control group participants were offered to join a one-month course with mindful eating workshops and YogaDance classes taking place after trial termination. Likewise, participants in the mindful eating and YogaDance groups were also offered the possibility of participating in the one-month course.

#### 2.4.2. Components of the Intervention

Each mindful eating workshop lasted 90 min. The program was centered around 10 eating principles (Supplementary Table S1). A workbook with exercises was handed out to participants so that they could train at home.

For YogaDance, the training intensity was selected to ensure participant adherence and meet the recommendations by the WHO for physical activity for adults (i.e., 150 min at moderate intensity or 75 min at high intensity per week). The first 20 min of a YogaDance class consisted of warming up and flow yoga. The warm-up included mindfulness meditation and gentle movements such as joint rotations, neck rolls, and side stretches. Subsequently, classic yoga poses and sequences were performed in a choreography to modern music in various genres (e.g., pop, electronica, hip-hop, rock). Some of the most utilized yoga poses in the first yoga part included warrior poses, downward facing dog, three-legged dog, cobra, cat-cow, plank pose, high and low lunges, boat pose and chair pose. When moving through the different poses and sequences, the movements happened on beat and in time with the music. The next 35 min of a YogaDance class consisted of dance, which was based on styles within Urban Dance (hip-hop, house, dancehall, and afro beat). During the dancing part, various dance steps and sequences were repeated and then put together as a choreography. Examples of steps used in YogaDance were “Bart Simpson”, “Azonto”, “Two-step”, “Coupe”, “Bogle move”, “Swing it away”, “Lock on”, and “Jack”. The last 20 min of a YogaDance class consisted of yoga, where the focus was on balance, stretching, and relaxation. In the balance part, poses such as tree pose, dancer pose, standing pigeon, warrior pose, and half-moon pose were presented. The YogaDance class continued with a relaxation part with gentle flows and stretches such as universal stretch, pigeon stretch, happy baby, hamstring stretch, and child’s pose. The YogaDance class ended with a short mindfulness meditation. It is worth mentioning that even though yoga has its roots in Hinduism and can be associated with a number of religious practices, in this study a non-religious, modern form of yoga was taught, exclusively with a focus on increasing physical and mental well-being.

Participants received 1–2 audio clips each week. The audio clips were adapted to each study group and served as supporting material for the different interventions. The audio clips for the YogaDance group included information about yoga and dance as well as the benefits of these two forms of movement. The audio clips for the mindful group included guided meditations, mindful eating exercises relevant to the mindful eating workshops and reflection exercises about eating behavior. The combined mindful eating and YogaDance group received both mindful eating and YogaDance audio clips. The control group received the same number of audio clips as the three active intervention groups, but the control clips were neutral and focused on general health advice on diet and exercise. The intention of the audio clips was also to keep participants motivated and ensure adherence.

### 2.5. Outcomes and Measurements

The primary outcome was fat mass. The secondary outcomes were body weight, BMI, waist circumference, fat percent, fat-free mass, visceral fat, bone mineral density, blood pressure, quality of life, eating behavior, energy intake, and physical activity. For all outcomes, measurements were recorded both at baseline and after the trial was completed (at 8 weeks).

#### 2.5.1. Anthropometry 

Height was only measured at the baseline. The participant was asked to remove shoes and to stand upright with the back to the wall-mounted stadiometer so that the back of the head, back, and buttock touched the stadiometer. The participant was further instructed to look straight ahead and to hold the arms relaxed, hanging next to the body. The height was recorded in centimeters to the nearest 0.5 cm.

Body weight and waist circumference were measured after the participant had emptied the bladder. Body weight was measured using a calibrated digital scale (Tanita WB-110MA). The participant was weighed in light clothes and asked to stand in the middle of the platform on the scale with a straight neck and eyes looking straight ahead while distributing the weight evenly on both feet. Body weight was recorded in kilograms to the nearest 0.1 kg.

The waist circumference was measured with the participant standing to ensure equal weight distribution on both feet. The waist circumference was measured twice on the skin with a non-elastic band to the nearest 0.5 cm between the lower rib and the hip crest, and when the participant exhaled. The average of these two measurements was used.

#### 2.5.2. Body Composition

Dual X-ray absorptiometry was used to measure fat mass, FFM, visceral fat, and BMD on a DXA scanner (Lunar iDXA with CoreScan module, GE Healthcare, Brøndby, Denmark). The scan was performed in a fasted state in the morning. The scan lasted approximately 10 min with the participant lying on her back on an open bed while an X-ray arm passed slowly across the body. Participants were asked not to wear metal objects on their bodies (e.g., jewelry, watches, hair clips, and zippers) and completed the scan in underwear or light clothing.

#### 2.5.3. Other Measurements

Systolic and diastolic blood pressures were measured using a validated automatic blood pressure monitor (UA-787 Plus) on the arms of participants that had rested 5–10 min in a sitting position prior to measuring. Measurements (recorded to the nearest 1 mmHg) were taken twice on both arms and averaged. In order to calculate calorie intakes, participants were also asked to record their diets for three days, before and after the intervention.

#### 2.5.4. Questionnaire Data: Quality of Life, Eating Behavior, and Physical Activity

All questionnaires were electronic and distributed to the participants via e-mail. SurveyXact (Rambøll Management Consulting, Aarhus, Denmark) was used for creating the questionnaires. The first questionnaire included the WHO-QOL-100 physical and mental dimensions, which examine a wide range of parameters related to quality of life including energy, fatigue, and sleep as well as self-esteem, body perception and positive/negative emotions [19]. The second questionnaire included the Intuitive Eating Scale-2, which examines emotional eating behavior as well as the perception of the body’s hunger and satiety signals [20]. For this study, the Intuitive Eating Scale-2 was translated from English to Danish and informally validated by asking approximately 10 randomly selected volunteers. Participants completed the IPAQ questionnaire on physical activity habits in everyday life and in leisure time [21]. Participants completed the three questionnaires at the baseline and again at the end of the trial.

#### 2.5.5. Compliance

All questionnaires were electronic and distributed to the participants via e-mail. SurveyXact Participation in YogaDance classes and mindful eating workshops was recorded by means of checklists (ticking off participants that showed up). Compliance was calculated for the participants who completed the trial.

### 2.6. Statistical Analysis

Descriptive statistics of baseline characteristics for participants within each intervention or the control group were obtained using medians and interquartile ranges.

Intention-to-treat (ITT) analyses were carried out based on all participants who were randomized. In the case of missing values, multiple imputation through chained equations was used based on 20 complete datasets. In this way, missing values were imputed using all variables in the datasets, exploiting correlations between variables to provide reasonable imputed values under the missing at random assumption [22]. ITT analyses provided results that may be interpreted as effects that would be seen in practice (“effectiveness”). In addition, complete-case analyses were carried out to obtain estimates that more closely reflected the best possible effects that may be achieved if participants adhered fully to the interventions (“efficacy”).

An analysis of covariance (ANCOVA) was used to compare mindful eating, YogaDance, and mindful eating and YogaDance combined to the control group (in total three pairwise comparisons) while including the baseline outcome level as a covariate. Additionally, ANCOVA models including both the baseline outcome and energy intake and physical activity at baseline as covariates were fitted. Model assumptions were assessed graphically by means of residual and QQ plots. Estimated mean differences between the three interventions and the control with corresponding 95% confidence intervals and *p*-values were reported (corresponding to three pairwise comparisons per outcome). For key results, estimated standardized effect sizes were also reported. The significance level was 0.05. Statistical analyses were conducted using the statistical software R (R Core Team, 2021). Multiple imputations were carried out using the R package “mice”.

## 3. Results

### 3.1. Study Progress

**Figure 1:**
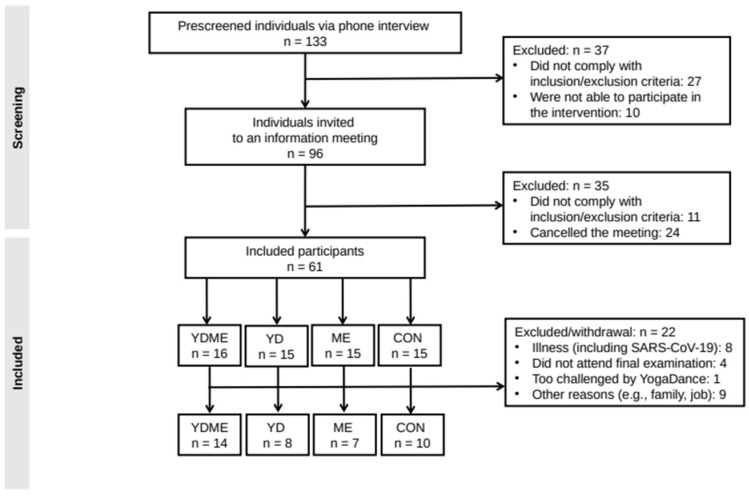
Flow chart of the study. CON = control, ME = mindful eating, YD = YogaDance, YDME = mindful eating and YogaDance combined.

A total of 133 persons were screened for eligibility, of whom 61 were found eligible for recruitment (from November 2021 to January 2022) and were randomized into one of the four groups after their baseline visit (Figure 1). Baseline measurements were taken in December 2021 and January 2022, and post-intervention measurements were taken from 30 March to 10 April 2022.

A total of 39 participants completed the trial, corresponding to a drop-out rate of 36% (Figure 1). Drop-out was unevenly distributed across intervention groups with 33%, 53%, 47%, and 13% in the control, mindful eating, YogaDance, and combined mindful eating and YogaDance groups, respectively. Reasons for drop-out appeared to be mostly unrelated to the interventions, supporting the missing at random assumption. Notably, 8 out of 22 drop-outs were related to illness including SARS-CoV-19 infections.

### 3.2. Baseline Characteristics

As expected, randomization resulted in small differences between the four intervention groups except for energy intake and physical activity where some medians differed by factors between 1.5 and 2 (Table 1). For the subgroup of completers (*n* = 39) there were also small differences between the four intervention groups although differences for some variables (including physical activity) were slightly larger than for the group of all randomized participants; there was no indication of selection bias (Supplementary Table S2). Overall, participant had a median age of 41 years (IQR: [28, 50] years), a median BMI of 27 kg/m^2^ (IQR: [26, 29] kg/m^2^), and a median waist circumference of 87 cm (IQR: [82, 92] cm).

### 3.3. Results of Intention-to-Treat Analyses

Fat mass was reduced by 1.3 kg (95% CI [−7.3, 10.0] kg; *p* = 0.77), 3.0 kg (95% CI [−5.1, 11.1] kg; *p* = 0.48), and 1.8 kg (95% CI [−6.6, 10.1] kg; *p* = 0.69) for the mindful eating, YogaDance, and combined mindful eating and YogaDance interventions, respectively (Table 2). These reductions corresponded to effect sizes of 0.15, 0.34, and 0.21 for the mindful eating, YogaDance, and combined mindful eating and YogaDance interventions, respectively. Likewise, for body weight, BMI, waist circumference, and systolic blood pressure decreases were seen in all three intervention groups compared to the control group. Fat-free mass, the two WHO quality of life scores, and energy intake increased for all three intervention groups compared to the control. Moreover, YogaDance tended to reduce anthropometric and body composition measures more than mindful eating, which in turn led to larger improvements in mental quality of life and eating behavior; the combination of mindful eating and YogaDance to some extent captured both types of improvements. Results did not change notably after adjustment for physical activity alone or both energy intake and physical activity (Supplementary Tables S3 and S4).

### 3.4. Results of Complete-Case Analyses

Fat mass was reduced by 1.0 kg (95% CI [−0.6, 2.6] kg; *p* = 0.23), 0.0 kg (95% CI [−1.6, 1.6] kg; *p* = 0.96), and 0.9 kg (95% CI [−0.4, 2.3] kg; *p* = 0.15) for the mindful eating, YogaDance, and combined mindful eating and YogaDance interventions, respectively (Table 3). These reductions corresponded to effect sizes of 0.11, 0.00, and 0.10 for the mindful eating, YogaDance, and combined mindful eating and YogaDance interventions, respectively. Fat percent, waist circumference, and systolic blood pressure were also reduced for all intervention groups compared to the control except for the combined mindful eating and YogaDance group, where fat percent was reduced by 1.1% points (95% CI [0.2, 2.1] % points; *p* = 0.02; effect size = 0.27). Fat-free mass, the two WHO quality of life scores, and the IES-2 total score increased for all three intervention groups compared to the control: WHO mental quality of life score (an increase of 1.3 on a scale from 4 to 20, 95% CI [0.7, 2.6]; *p* = 0.04; effect size = 0.57) and the IES-2 total score (an increase of 0.4 on a scale from 1 to 5, 95% CI [0.1, 0.7; *p* = 0.01]; effect size = 0.73). Furthermore, there was an increase in the IES-2 total score of 0.5 (95% CI [0.1, 0.9; *p* = 0.01]; effect size = 0.91) for the mindful eating group. It should also be noted that anthropometric outcomes were in general improved more for the mindful eating group than for the YogaDance group. Results were almost unaltered after adjustment for physical activity (Supplementary Table S5) but additional adjustment for energy intake led to slightly larger improvements for anthropometry outcomes for the mindful eating group compared to the YogaDance group: waist circumference (an increase of −4.6 cm, 95% CI [−8.9, −0.3] cm; *p* = 0.04; effect size = 0–60) (Supplementary Table S6).

### 3.5. Compliance

Overall, compliance was low to moderate. For the mindful eating group compliance declined over time: 100%, 89%, 44%, and 44%. For the YogaDance group, compliance fluctuated randomly across the 8-week intervention period: 100% (first week), 75%, 71%, 83%, 79%, 75%, 69%, and 71% (last week). Likewise, for the combined mindful eating and YogaDance group, the pattern in the compliance also fluctuated randomly across the 8 weeks: 79% (first week), 64%, 86%, 71%, 86%, 76%, 82%, and 71% (last week). In addition, a total of 24 YogaDance classes were planned, but only 22 were held, as two classes were canceled due to the illness of the instructors.

## 4. Discussion

Mindful eating and the combination of mindful eating and YogaDance showed more improvements than YogaDance alone. The estimated effect sizes were small, at most 0.11 and 0.35 in complete-case and intention-to-treat analyses, respectively, in any case far smaller than the anticipated effect size used for the sample size calculation. However, notably for the combined mindful eating and YogaDance group, but also for the mindful eating group alone, there were a few results for several secondary outcomes, showing a small reduction in fat percent and waist circumference as well as improved eating behavior and mental quality of life. These results were only found in complete-case analyses, implying that they could cautiously be interpreted as efficacy estimates in the event that participants adhered fully to the intervention.

As this study investigated the effect of combining mindful eating and YogaDance on fat mass, there is not much comparable evidence in the literature. However, some relevant results for other body composition measures exist for the individual effects of yoga, dance, and mindful eating interventions [23,24]. This study found reductions in body weight, fat percent, BMI, and waist circumference of similar magnitudes as seen previously for yoga also for an 8-week intervention [15]. Moreover, another systematic review and meta-analysis on dance found that dance interventions decreased body weight, BMI, and fat mass [25]. A systematic review and meta-analysis investigated the effect of the popular exercise program Zumba^®^, which has some similarities to YogaDance and aims at several of the same health benefits (physical fitness, weight and fat loss, well-being and improved quality of life), and found a reduction in fat mass of 0.3% [26], a reduction very similar to the ones found in the present study for the ITT analysis for the combined mindful eating and YogaDance group. This study also found an improvement in the mental quality of life score in the combined mindful eating and YogaDance group. Several studies on yoga-related interventions have also demonstrated improved quality of life and well-being [27,28,29,30] for a range of scales including one version of the WHO-QOL.

A randomized controlled trial on mindful eating in overweight women also found improved eating behavior but no effects on body weight, waist circumference, fat mass, or fat-free mass [31]. In this study only a small reduction in weight were found; such reductions have also been observed in a previous study [32]. A recent systematic review and meta-analysis found no effect on energy intake, as was also the case in this study [33]. In this study mindful eating alone and in combination with YogaDance led to improvements in eating behavior. This finding agrees with two previous studies that involved 8 weeks and 4 months of intervention, respectively, and that also found improvements in eating behavior for a range of behavioral scales, including several subscales of the Dutch Eating Behaviour Questionnaire (DEB-Q) [34,35], but not for IES-2.

This study has some strengths. Firstly, to our knowledge, this is the first randomized controlled trial to investigate a novel exercise approach that combines yoga, dance, and mindful eating. Secondly, the study provided a comprehensive investigation through the use of a wide range of outcomes including anthropometric and body composition outcomes as well as physical activity, quality of life, and eating behavior.

The study has several limitations. The large drop-out, which is unevenly distributed across intervention groups, is a key limitation that may have led to selection bias. It seemed that participants preferred YogaDance over mindful eating. One explanation could be that the trial coincided partly with the last lockdown period in Denmark due to the SARS-CoV-19 pandemic. The trial was initiated during the lockdown, but most restrictions had been lifted when the trial terminated. People may have preferred activities that involved more physical activity. The sample size would have needed to accommodate a higher drop-out rate (at least 40%) to ensure that sufficient participants would eventually complete the trial. However, it should be noted that the drop-out rate in this study is no different from what has been seen in the past for weight management trials [36,37].

Low compliance, which was different between intervention groups, is another key limitation as it may have introduced a selection bias. Moreover, it may dilute the effects of the interventions. It would have been useful to supervise daily training activities remotely, for instance, through online video meetings, to ensure high compliance. A too-low training load may also have diluted the benefits of the interventions. Specifically, a total of four mindful eating workshops with a duration of only 90 min and unsupervised daily training based on handout workbooks might have resulted in a low training intensity. It was a compromise, as participants were encouraged to do exercises at home as well. Monitoring participants’ training loads by means of %HR max, MET, or %VO_2_ peak would have enabled a more precise assessment of individual training loads, and it would also have allowed to adjust for differences in compliance between participants, which was not possible in the present study.

There are also some additional limitations. The wide range used for some inclusion criteria such as age, BMI and waist circumference introduced a large between-participant heterogeneity, making it much more difficult to observe large effects. Moreover, the effect of YogaDance depended highly on each participant’s ability and technical skills, which for many participants were low due to excess body weight and a history of inactivity. The significant results obtained in the complete-case analyses should also be interpreted cautiously, as they are based on a reduced, random subgroup of participants and therefore selection bias cannot be ruled out (as randomization was broken), although the reasons for missing data seem to render complete-case analyses meaningful [38]; significant results from such a small randomized trial should be interpreted carefully and in combination with effect estimates. Moreover, *p*-values were not adjusted for multiplicity, implying that the risk of false positive results is slightly elevated above 5%. Finally, it should be mentioned that the Danish version of the IES-2 scale was not formally validated.

## 5. Conclusions

The study found a modest reduction in fat percent as well as modest improvements in quality of life and eating behavior. The practical implication is that the combination of mindful eating and YogaDance may be an accessible and feasible intervention for overweight and obese women. However, the study had low power to establish benefits of the intervention due to several key shortcoming, notably a large drop-out rate and low compliance.

## Figures and Tables

**Table 1:** Baseline participant characteristics stratified according to intervention group.

	Mindful Eating + YogaDance	YogaDance	Mindful Eating	Control
	(*n* = 16)	(*n* = 15)	(*n* = 15)	(*n* = 15)
Age, years	37 (29–48)	44 (32–50)	45 (35–56)	42 (32–54)
Height, m	1.66 (1.63–1.70)	1.67 (1.64–1.72)	1.72 (1.69–1.74)	1.69 (1.65–1.73)
FM, kg	31.7 (25.5–35.8)	33.4 (29.6–40.0)	39.6 (31.9–48.9)	35.2 (27.5–42.6)
FFM, kg	42.5 (40.0–45.2)	43.1 (38.8–47.4)	47.1 (44.2–47.7)	46.0 (43.4–49.5)
BW, kg	78.1 (70.0–84.0)	79.5 (73.6–87.1)	87.9 (75.6–95.3)	83.7 (74.6–94.8)
Fat percent, %	41 (38–44)	44 (39–46)	43 (39–46)	42 (38–47)
BMI, kg/m^2^	27 (26–30)	28 (26–30)	29 (27–33)	28 (27–34)
WC, cm	89 (82–95)	86 (85–94)	94 (85–99)	93 (88–103)
VF, g	788 (575–1323)	797 (633–1247)	1047 (607–1324)	1156 (752–1240)
BMD, g/cm^2^	1.2 (1.2–1.3)	1.2 (1.2–1.3)	1.2 (1.2–1.3)	1.3 (1.2–1.3)
SBP, mmHg	106 (97–114)	112 (104–122)	111 (105–117)	111 (105–125)
DBP, mmHg	72 (66–77)	74 (70–80)	74 (69–80)	75 (69–83)
WHO-QOL physical dimension	14 (13–15)	12 (11–13)	13 (10–16)	15 (14–16)
WHO-QOL mental dimension	13 (12–15)	12 (11–14)	12 (12–15)	14 (14–15)
IES-2 total	2.9 (2.8–3.5)	2.7 (2.6–3.3)	3.2 (2.6–3.6)	3.4 (2.8–3.7)
EI, kcal/day	1994 (1261–2411)	1052 (499–1440)	1533 (1122–2894)	1477 (983–3345)
MET, min/week	1436 (958–1967)	838 (353–1125)	900 (790–2389)	1234 (715–2536)

Data are shown as medians and interquartile ranges. BMD = bone mass density, BMI = body mass index, BW = body weight, DBP = diastolic blood pressure, EI = energy intake, FFM = fat-free mass, FM = fat mass, IES-2 = Overall Intuitive Eating Scale-2 score (values in the interval 1–5) MET = metabolic equivalents of task, SBP = systolic blood pressure, VF = visceral fat, WC = waist circumference, WHO-QOL = World Health Organization quality of life score (values in the interval 4–20).

**Table 2:** Pairwise comparisons of means at the end of the study (after 8 weeks) based on unadjusted intention-to-treat analyses (*n* = 61).

	Mindful Eating vs. Control		YogaDance vs. Control		Mindful Eating + YogaDance vs. Control	
	(*n* = 15 vs. *n* = 15)		(*n* = 15 vs. *n* = 15)		(*n* = 16 vs. *n* = 15)	
	Estimate	*p*-Value	Estimate	*p*-Value	Estimate	*p*-Value
**Outcome**						
FM, kg	−1.3 (−10.0, 7.3)	0.77	−3.0 (−11.1, 5.1)	0.48	−1.8 (−10.1, 6.6)	0.69
FFM, kg	1.4 (−3.5, 6.2)	0.58	2.5 (−2.9, 7.8)	0.37	0.5 (−5.0, 6.1)	0.86
BW, kg	−0.8 (−16.7, 15.9)	0.92	−3.3 (−17.4, 10.8)	0.65	−2.4 (−17.1, 12.3)	0.76
Fat percent, %	0.4 (−3.2, 4.0)	0.83	−2.1 (−7.0, 2.7)	0.41	−0.4 (−3.8, 3.0)	0.81
BMI, kg/m^2^	−0.6 (−2.9, 1.7)	0.60	−0.1 (−2.7, 2.5)	0.93	−0.2 (−3.0, 2,6)	0.90
WC, cm	−0.4 (−9.3, 8.5)	0.93	−2.2 (−13.5, 9.1)	0.72	−3.0 (−12.0, 6.0)	0.52
VF, g	−107 (−492, 278)	0.59	−97 (−497, 303)	0.64	2 (−359, 364)	0.99
BMD, g/cm^2^	−0.03 (−0.15, 0.09)	0.67	−0.02 (−0.12, 0.09)	0.72	0.00 (−0.09, 0.09)	0.98
SBP, mmHg	−7.9 (−20.0, 4.3)	0.21	−7.8 (−19.9, 4.2)	0.21	−1.3 (−13.8, 11.1)	0.83
DBP, mmHg	−1.4 (−10.2, 7.5)	0.77	−0.3 (−9.0, 8.3)	0.94	0.7 (−7.9, 9.2)	0.88
WHO-QOL physical dimension	0.5 (−2.8, 3.8)	0.76	1.2 (−1.7, 4.0)	0.44	1.0 (−1.6, 3.6)	0.46
WHO-QOL mental dimension	1.6 (−2.3, 5.4)	0.46	1.3 (−2.2, 4.8)	0.49	1.2 (−1.9, 4.3)	0.46
IES-2 total	0.3 (−0.3, 0.9)	0.38	−0.04 (−0.6, 0.5)	0.90	0.4 (−0.1, 0.8)	0.17
EI, kcal/day	576 (−3492, 4646)	0.79	1062 (−2855, 4978)	0.60	598 (−3623, 4820)	0.79
MET, min/week	406 (−2503, 3315)	079	−258 (−4074, 3559)	0.90	9.9 (−3664, 3684)	>0.99

Data are shown as mean differences with corresponding 95% confidence intervals and *p*-values based on analysis of covariance including the baseline outcome value as covariate. BMD = bone mass density, BMI = body mass index, BW = body weight, DBP = diastolic blood pressure, EI = energy intake, FFM = fat-free mass, FM = fat mass, IES-2 = Overall Intuitive Eating Scale-2 score (values between 1 and 5) MET = metabolic equivalents of task, SBP = systolic blood pressure, VF = visceral fat, WC = waist circumference, WHO-QOL = World Health Organization quality of life score (values in the interval 4–20).

**Table 3:** Pairwise comparisons of means at the end of the study (after 8 weeks) based on unadjusted complete-case analyses (*n* = 39).

	Mindful Eating vs. Control		YogaDance vs. Control		Mindful Eating + YogaDance vs. Control	
	(*n* = 7 vs. *n* = 10)		(*n* = 8 vs. *n* = 10)		(*n* = 14 vs. *n* = 10)	
	Estimate	*p*-Value	Estimate	*p*-Value	Estimate	*p*-Value
FM, kg	−1.0 (−2.6, 0.6)	0.23	0.0 (−1.5, 1.6)	0.96	−0.9 (−2.3, 0.4)	0.16
FFM, kg	0.6 (−0.6, 1.9)	0.32	0.1 (−1.1, 1.3)	0.88	0.5 (−0.6, 1.6)	0.35
BW, kg	−1.0 (−3.2, 1.2)	0.35	0.2 (−1.9, 2.4)	0.82	−0.2 (−2.1, 1.6)	0.81
Fat percent, %	−0.6 (−1.7, 0.6)	0.33	−0.1 (−1.2, 1.0)	0.86	−1.1 (−2.1, −0.2)	0.02
BMI, kg/m^2^	−0.3 (−1.1, 0.4)	0.41	0.2 (−0.6, 0.9)	0.62	−0.03 (−0.6, 0.6)	0.93
WC, cm	−3.9 (−8.0, 0.3)	0.06	−0.8 (−4.9, 3.4)	0.72	−3.4 (−6.9, 0.1)	0.06
VF, g	−78 (−206, 500)	0.22	63 (−64, 190)	0.32	−36 (−144, 71)	0.49
BMD, g/cm^2^	−0.00 (−0.03, 0.03)	0.82	−0.00 (−0.03, 0.03)	0.89	0.00 (−0.02, 0.03)	0.83
SBP, mmHg	−3.5 (−12.9, 6.0)	0.46	−3.7 (−12.6, 5.2)	0.40	−0.7 (−8.8, 7.4)	0.86
DBP, mmHg	−1.0 (−6.9, 4.8)	0.72	1.7 (−3.9, 7.2)	0.55	1.9 (−3.0, 6.8)	0.43
WHO-QOL physical dimension	1.1 (−0.6, 2.8)	0.18	0.8 (−0.9, 2.5)	0.34	1.1 (−0.3, 2.5)	0.13
WHO-QOL mental dimension	1.2 (−0.3, 2.7)	0.10	0.3 (−1.2, 1.8)	0.69	1.3 (0.7, 2.6)	0.04
IES-2 total	0.5 (0.1, 0.9)	0.01	−0.02 (−0.4, 0.4)	0.93	0.4 (0.1, 0.7)	0.01
EI, kcal/day	−559 (−3886, 2769)	0.74	894 (−2261, 4049)	0.57	885 (−1823, 3594)	0.51
MET, min/week	−370 (−2629, 1889)	0.74	212 (−1965, 2390)	0.84	612 (−1250, 2473)	0.51

Data are shown as mean differences with corresponding 95% confidence intervals and *p*-values based on analysis of covariance including the baseline outcome value as covariate. BMD = bone mass density, BMI = body mass index, BW = body weight, DBP = diastolic blood pressure, EI = energy intake, FFM = fat-free mass, FM = fat mass, IES-2 = Overall Intuitive Eating Scale-2 score (values in the interval 1–5) MET = metabolic equivalents of task, SBP = systolic blood pressure, VF = visceral fat, WC = waist circumference, WHO-QOL = World Health Organization quality of life score (values in the interval 4–20).

## Data Availability

Raw data will be made available upon reasonable request.

## Supplementary Materials

The following supporting information can be downloaded at: https://www.mdpi.com/article/10.3390/nu15071646/s1, Table S1 provides details on the mindful eating intervention. Tables S2–S6 provide baseline characteristics for completers, results for intention-to-treat and complete-case analyses, respectively, adjusted for either physical activity alone or both energy intake and physical intake.

## Abbreviations

BMI = body mass index; BMD = bone mass density; BMI = body mass index; BW = body weight; CON = control; DBP = diastolic blood pressure; EI = energy intake; FFM = fat-free mass; FM = fat mass; HR max = maximum heart rate; IES-2 = Overall Intuitive Eating Scale-2 score (values in the interval 1–5); IQR = interquartile range; ME = mindful eating; MET = metabolic equivalents of task; SBP = systolic blood pressure; VF = visceral fat; VO_2_ max/peak = maximum/peak oxygen uptake; WC = waist circumference; WHO-QOL = World Health Organization quality of life score (values in the interval 4–20); YD = YogaDance; YDME = mindful eating and YogaDance combined.
