# Supplementary material for: Effects of Mindful Eating and YogaDance among Overweight and Obese Women: An Exploratory Randomized Controlled Trial
Source: Nutrients. 2023 Mar 28;15(7):1646. doi: 10.3390/nu15071646 (PMC10096929; doi:10.3390/nu15071646)
Supplement: Supplementary file 1 [file nutrients-15-01646-s001.zip › suppl-tables-S1.pdf]

Table S1. Overview of mindful eating workshops.

**Workshop**	**Theme**	**Content**
#1	Introduction to the 10 principles<br><br>Principle 1+2+3	*Introduction (25 min):*<br>● *Check-in exercise: Body scan.*<br>● *Introduction to the 10 principles*<br><br>*Main body (55 min):*<br>● *Principle 1: Information about the Danish dietary guidelines and a plenum discussion about pros and cons*<br>● *Principle 2: Exercise about "forbidden" foods and group discussion*<br>● *Principle 3: Exercise with illustration of healthy meals that include the 5 basic tastes and satisfy the senses.*<br><br>*End (10 min)*<br><br>*Check-out exercise: word of the day.*
#2	Principle 4+5+6	*Introduction (15 min):*<br>● *Check-in exercise: Body scan.*<br><br>*Main body (65 min):*<br>● *Principle 4: Information and exercise about nudging in relation to food and eating behavior.*<br>● *Principle 5: Mindful eating exercise ("the raisin exercise").*<br>● *Principle 6: Positive affirmation exercise.*<br><br>*End (10 min)*<br><br>*Check-out exercise: word of the day.*
#3	Principle 7	*Introduction (15 min):*<br>● *Check-in exercise: Body scan.*<br><br>*Main body (65 min):*<br>● *Principle 7: Walk and talk in Frederiksberg Have about how to move with joy and pleasure. Small exercises during the walk.*<br><br>*End (10 min)*<br><br>*Check-out exercise: word of the day.*
#4	Principle 8+9+10	*Introduction (15 min):*<br>● *Check-in exercise: Body scan.*<br><br>*Main body (55 min):*<br>● *Principle 8: Dinner with a shared*

buffet (everyone brings a small meal to share)

- *Principle 9: Pair exercise with focus on self-love and body acceptance*
- *Principle 10: "Speed-dating" with sharing hobbies and interests. Joint ending where we sing a song together.*

End (20 min)

Check-out exercise: word of the day.

Mindful eating workshop 1

The first part of the workshop consisted of an introduction to the 10 principles. The participants worked with the first three principles that introduced the official Danish dietary guidelines, the concept of mindful eating, methods and exercises to listen to the body's hunger and satiety signals, how to allow all foods in a balanced way as well as how to create meals that include the five basic tastes (sour, sweet, bitter, salty, umami) and satisfy the senses.

Mindful eating workshop 2

In the second workshop, the participants worked with the principles 4, 5 and 6. They were introduced to the concept of "nudging" and how to use this tool to improve eating behavior and make healthier eating choices. The participants were also introduced to a mindful eating exercise known as "the raisin exercise" aimed at creating more awareness of the food while eating. Finally, the participants worked with positives affirmations (e.g. "I love and respect myself and my body", "I am worthy of love"), which can help improve self-esteem and body acceptance.

Mindful eating workshop 3

The third workshop focused on principle 7 and took place in a garden in Copenhagen. The participants did a "walk and talk" together in small groups where they were encouraged to reflect on and discuss their motivation towards physical activity and what is considered enjoyable movement for them.

Mindful eating workshop 4

In the last workshop, the participants worked with principle 8, 9 and 10. They brought a small dish to contribute to a shared buffet. While eating, a discussion about the benefits of sharing food was initiated. Hereafter, participants did a pair exercise that targeted cultivating self-love and self-acceptance. In the last part of the workshop, the participants had to share their hobbies and interests, and a discussion about what constitutes a good and meaningful life was facilitated. The workshop ended with everyone singing a song together.

The 10 principles

In the following section, the 10 principles are presented. The principles are described in the same way as they were presented to the study participants, i.e. in lay language.

1. Eat for your health and not for your weight

In Denmark we have official dietary guidelines that are based on solid research on how to prevent obesity and non-communicable diseases. Find inspiration in these guidelines and try to eat vegetables, fruit and wholegrain on a daily basis while limiting meat, sugar and saturated fat consumption. Eat a variety of foods so you ensure yourself enough vitamins and minerals. But most importantly – think of them as guidelines and not as another strict diet plan that you should follow word-by-word. If you can get

some vegetables, fruit and wholegrain into the diet on a daily basis, that's great! Otherwise, start slower (for example every other day) and build up from there.

2. Allow all foods and stop counting calories

If you have been avoiding certain foods and banned yourself from eating more than a certain amount of calories each day, this principle will be difficult to adhere to at first. But it cannot be stressed enough how important this principle is to practice; once you get there, you will see why. The diet mentality, where certain foods are allowed while others are forbidden, will keep you in the "diet jail" where all of your thoughts revolve around food. When you restrict calories for a long period of time (as you do when you are on a diet), your metabolism decreases in order to save some of the limited available energy. Furthermore, it will make your brain constantly hunt high calorie foods to keep you alive. In short, when you go on a diet, your body actually thinks you are in danger and will try to save you! Do you see how this makes it almost impossible to win against biology? By allowing all foods and stopping the eternal calorie counting, you will shift your focus from external to internal cues to eat. In other words, you will begin listening to what and how much energy your body actually needs - and you will begin cooperating with biology, which is much better and easier than fighting it.

3. Satisfy your senses and feed your taste buds

Do you know the feeling of "missing" something after completing a meal? Most often something sweet? Not including all the five basic tastes in a meal can create a feeling of not being completely satisfied even though you are physically full. Therefore, try to get sweet, bitter, sour, salty and umami into your main meals. Also, use your senses when you eat; smell the food, create meals with beautiful colors and combine your meals with foods that are both soft and crunchy as well as hot and cold. Of course, this is not possible to do every time you eat - and you shouldn't! The most important aspect of this principle is to start noticing what tastes, textures and flavors you desire in a given moment in order to give you the highest possible satisfaction every time you eat. Try it out, play with it and notice the difference it makes in your feeling of satiation - and satisfaction.

4. Make healthy living easy and cultivate good habits

Remember that you are just a human! The evolution of the human brain has not kept up with the evolution of modern society. The human brain is all about securing enough energy for difficult times in the future, but it's not aware that food will never be a limiting factor - at least not in our modern Western societies. With food being available almost everywhere and with trains, busses and cars helping us move around, it's more difficult to actively make healthier choices. You need to trick your primal brain and you can do this by simple strategies, e.g. choosing smaller plate sizes, taking a detour if there lies a tempting bakery on the way home, avoid shopping when hungry and write a shopping list beforehand. By doing these - and other - simple tricks, you can nudge yourself to eat and live healthier. Another important thing to work on is habits. Maybe you've made a habit of eating in front of the television or to snack before dinner? Habits can be difficult to alter, but by cultivating good habits, you can achieve many health benefits for the rest of your life.

5. Eat when you eat and take your time

When you eat, try not to engage in any distractions such as your smartphone or television. This can be hard at first - especially if you eat alone - but it will get better with practice. Make sure that you eat in a calm and pleasant environment (maybe lighting some candles and putting on some quiet, calming music) and try to eat slowly in order to feel your fullness. Take a deep breath before, during and after your meal to help the body and mind calm down. Following this principle will help you to better notice your hunger and satiety signals.

6. Listen to your body's needs and take care of yourself

This is all about paying close attention to what your body actually needs - and it can be a real challenge to listen if you have been used to giving it strict orders. Some of the questions you can ask yourself to become a better listener are: What do I really desire

to eat - something fresh, sweet, salty, crunchy? Am I actually truly hungry or do I need something other than food - a talk, a walk or a hug? Am I hungry or maybe just thirsty? If you tend to be an emotional eater, e.g. if you eat when you feel restless, lonely, bored, sad or stressed, this principle is crucial for you. In this case, try to figure out what needs or emotions you use food to cope with and instead begin to meet those needs with self-care and respect - and without using food.

7. *Move with joy and pleasure*

Physical activity is healthy and prevents various lifestyle diseases. However, you may feel that exercising and training are a tedious obligation that must be overcome. For many people, exercise is not associated with either pleasure or enjoyment. Nevertheless, moving your body can help you deal with negative thoughts about food and your own body. Therefore, we encourage you to exercise in a way that is enjoyable, pleasurable and motivating for you. Maybe you love walking in nature or maybe dancing is what makes your body sing. If you haven't found your "thing" yet, then try out different forms of movement - and when you find something that you feel like doing again and again, then stick with it!

8. *Share food with others and feel the difference*

Humans are social beings; when we share food, it is much easier to eat healthily and enjoy the food in good company. When we dine together, it's a lot easier to slow down the eating speed and thus pay attention to our bodily sensations. Moreover, sharing food can be magical because you enjoy the eating experience together. When you cook for others - or someone cooks for you - it can almost feel like a gift of love. Cooking is also a wonderful way of spending time together - and the mealtime will then feel like harvesting the fruits of a shared work. If you have problems with eating too little or too much, eating with others can help you regulate this behavior and get the amount of food that you actually need.

9. *Be the change and break the taboos*

A study from the Danish Ethics Council shows that the ideal body is perceived by many as slim and toned. This principle is about breaking down body ideals and accepting that all bodies are different. In order to break down this taboo, first of all it is necessary not to comment on the weight of others. It is not the individual's own fault whether they are underweight or overweight, as there are many factors that play a role in whether one becomes overweight or not. However, to break down the anxiety of gaining weight, it is important to talk about and articulate your thoughts and feelings related to your body weight and shape, since these can be destructive to your attempts to adopt healthy eating behaviors. It can be a great help to find out that you are not the only one who has a difficult relationship with food and your body. Therefore, be brave and share your thoughts and feelings with others - there is a good chance that they will resonate. In short, be the change, break the taboos and let's start a movement together, where it's not shameful to talk openly about body image and difficult eating behaviors.

10. *Fill your life with purpose and happiness*

Our relationship with food, exercise and our own body has a lot to do with our feeling of purpose and happiness in life. If you are passionate about something - this could be playing guitar, knitting sweaters, being politically active, doing drawings or being in your garden - you will simply care less about how your body appears. Cultivating a hobby is a healthy way to unwind and forget the hustle-and-bustle of everyday life and it can allow you to immerse yourself and become good at something - and that, in itself, can be hugely satisfying. Find something that interests you and sign up solo or as a team. On teams, there is the opportunity to meet others who share the same interests, which will give you the opportunity to create new relationships. A new hobby or interest can be a good way to divert thoughts and frustrations about food and your body. Also, laugh more, love more and enjoy more! For a moment, just think about all the wonderful stuff you could do for yourself, your loved ones and your surroundings if you stopped spending your time and energy worrying about what you eat and how your body appears.
