# Supplementary material for: Effects of Mindful Eating and YogaDance among Overweight and Obese Women: An Exploratory Randomized Controlled Trial
Source: Nutrients. 2023 Mar 28;15(7):1646. doi: 10.3390/nu15071646 (PMC10096929; doi:10.3390/nu15071646)
Supplement: Supplementary file 1 [file nutrients-15-01646-s001.zip › suppl-tables-S2-S6.pdf]

Table S2. Baseline participant characteristics stratified according to intervention group for completers only (n=39).

	**Mindful eating +YogaDance (n=14)**	**YogaDance (n=8)**	**Mindful eating (n=7)**	**Control (n=10)**
Age, years	39 (28-49)	41 (34-46)	45 (27-56)	41 (31-52)
Height, m	1.67 (1.64-1.72)	1.68 (1.63-1.74)	1.74 (1.71-1.75)	1.68 (1.64-1.72)
FM, kg	31.7 (24.8-34.9)	31.0 (24.6-33.6)	32.3 (31.9-40.3)	30.5 (27.3-37.1)
FFM, kg	41.9 (39.9-44.0)	44.2 (41.0-47.5)	47.3 (44.3-50.6)	45.3 (42.6-48.4)
BW, kg	73.8 (68.7-82.6)	77.2 (73.7-80.0)	87.9 (80.4-89.9)	78.5 (73.5-86.2)
Fat percent, %	41 (38-43)	39 (36-44)	43 (39-45)	40 (36-45)
BMI, kg/m^2	27 (26-28)	27 (26-28)	29 (28-30)	27 (26-31)
WC, cm	87 (82-91)	85 (80-85)	91 (87-94)	90 (81-99)
VF, g	761 (533-1283)	634 (483-703)	1032 (658-1211)	1034 (383-1204)
BMD, g/cm^2	1.2 (1.2-1.3)	1.2 (1.2-1.3)	1.3 (1.2-1.3)	1.3 (1.2-1.3)
SBP, mmHg	106 (97-113)	113 (104-118)	106 (102-109)	109 (104-124)
DBP, mmHg	72 (65-78)	73 (68-79)	72 (69-74)	74 (69-80)
WHO-QOL physical dimension	14 (14-15)	12 (11-14)	13 (13-17)	15 (14-16)
WHO-QOL mental dimension	13 (12-15)	12 (12-14)	15 (14-16)	14 (14-15)
IES-2 total	2.9 (2.84-3.5)	2.8 (2.5-3.2)	3.0 (2.6-3.6)	3.4 (3.2-3.7)
EI, kcal/day	1994 (1400-2388)	816 (559-1088)	2503 (1694-3657)	1490 (1179-2657)
MET, min/week	1436 (1037-2062)	692 (428-855)	1788 (1190-2665)	1262 (883-2131)

Data shown as medians and interquartile ranges.

BMD=bone mass density, BMI=body mass index, BW=body weight, DBP=diastolic blood pressure, EI=energy intake, FFM=fat-free mass, FM=fat mass, IES-2=Overall Intuitive Eating Scale-2 score (values in the interval 1-5), MET=metabolic equivalents of task, SBP=systolic blood pressure, VF=visceral fat, WC=waist circumference, WHO-QOL=World Health Organization quality of life score (values in the interval 4-20).

Table S3. Pairwise comparisons of means at end of study (after 8 weeks) based on intention-to-treat analyses adjusted for physical activity (n=61).

	**Mindful eating vs. control (n=15 vs n=15)**		**YogaDance vs. control (n=15 vs n=15)**		**Mindful eating+YogaDance vs. control (n=16 vs n=15)**	
	Estimate	p-value	Estimate	p-value	Estimate	p-value
**Outcome**						
FM, kg	-1.6 (-10.2, 7.0)	0.73	-2.5 (-10.6, 5.6)	0.56	-1.8 (-10.0, 6.6)	0.68
FFM, kg	1.3 (-3.6, 6.1)	0.61	2.7 (-2.6, 8.1)	0.32	0.4 (-5.1, 6.0)	0.88
BW, kg	-0.9 (-16.8, 15.0)	0.91	-3.0 (-17.1, 11.1)	0.69	-2.5 (-17.2, 12.2)	0.75
Fat percent, %	0.3 (-3.2, 3.9)	0.86	-2.0 (-6.8, 2.9)	0.46	-0.5 (-3.9, 3.0)	0.80
BMI, kg/m^2	-0.7 (-3.0, 1.7)	0.58	0.00 (-2.6, 2.6)	>0.99	-0.2 (-3.0, 2.6)	0.89
WC, cm	-0.3 (-9.2, 8.6)	0.95	-2.5 (-13.8, 8.8)	0.67	-3.0 (-12.0, 6.0)	0.53
VF, g	-110 (-494, 275)	0.59	89 (-490, 312)	0.69	2 (-360, 364)	0.99
BMD, g/cm^2	-0.03 (-0.15, 0.09)	0.65	-0.02 (-0.12, 0.09)	0.77	0.00 (-0.09, 0.09)	>0.99
SBP, mmHg	-8.0 (-20.1, 4.2)	0.21	-7.5 (-19.5, 4.6)	0.26	-1.4 (-13.9, 11.1)	0.83
DBP, mmHg	-1.2 (-10.0, 7.7)	0.80	-0.9 (-9.6, 7.7)	0.84	0.8 (-7.8, 9.3)	0.86
WHO-QOL physical dimension	0.4 (-2.9, 3.7)	0.81	1.4 (-1.5, 4.3)	0.36	0.9 (-1.6, 3.5)	0.48
WHO-QOL mental dimension	1.5 (-2.4, 5.3)	0.50	1.4 (-2.1, 4.9)	0.45	1.1 (-1.9, 4.2)	0.49
IES-2 total	0.3 (-0.3, 0.9)	0.39	-0.02 (-0.6, 0.6)	0.95	0.3 (-0.1, 0.8)	0.17
EI, kcal/day	21 (-4048, 4090)	0.99	595 (-3320, 4511)	0.76	1425 (-2797, 5646)	0.50

Data shown as mean differences with corresponding 95% confidence intervals and p-values based on analysis of covariance including the baseline outcome value and baseline physical activity as covariates.

BMD=bone mass density, BMI=body mass index, BW=body weight, DBP=diastolic blood pressure, EI=energy intake, FFM=fat-free mass, FM=fat mass, IES-2=Overall Intuitive Eating Scale-2 score (values in the interval 1-5), SBP=systolic blood pressure, VF=visceral fat, WC=waist circumference, WHO-QOL=WHO quality of life score (values in the interval 4-20).

Table S4. Pairwise comparisons of means at end of study (after 8 weeks) based on intention-to-treat analyses adjusted for both energy intake and physical activity (n=61).

	**Mindful eating vs. control (n=15 vs n=15)**		**YogaDance vs. control (n=15 vs n=15)**		**Mindful eating+YogaDance vs. control (n=16 vs n=15)**	
	Estimate	p-value	Estimate	p-value	Estimate	p-value
**Outcome**						
FM, kg	-1.6 (-10.2, 7.0)	0.72	-2.8 (-10.9, 5.3)	0.52	-1.6 (-9.9, 6.8)	0.72
FFM, kg	1.2 (-3.7, 6.1)	0.64	2.6 (-2.7, 8.0)	0.33	0.5 (-5.1, 6.0)	0.88
BW, kg	-1.2 (-17.1, 14.7)	0.89	-3.6 (-17.7, 10.5)	0.64	-2.5 (-17.2, 12.3)	0.75
Fat percent, %	0.3 (-3.3, 3.9)	0.88	-2.0 (-6.8, 2.9)	0.46	-0.4 (-3.8, 3.0)	0.83
BMI, kg/m^2	-0.7 (-3.0, 1.7)	0.59	0.01 (-2.6, 2.6)	>0.99	-0.2 (-3.0, 2.6)	0.89
WC, cm	-0.5 (-9.5, 8.4)	0.91	-2.7 (-14.0, 8.6)	0.66	-2.9 (-11.9, 6.2)	0.55
VF, g	-122 (-507, 263)	0.55	98 (-498, 303)	0.66	15 (-347, 378)	0.94
BMD, g/cm^2	-0.03 (-0.15, 0.09)	0.65	-0.02 (-0.12, 0.09)	0.77	0.00 (-0.09, 0.09)	0.98
SBP, mmHg	-7.9 (-20.1, 4.3)	0.24	-7.4 (-19.5, 4.6)	0.27	-1.5 (-13.9, 11.0)	0.82
DBP, mmHg	-1.2 (-10.0, 7.7)	0.79	-0.9 (-9.6, 7.7)	0.84	0.8 (-7.7, 9.3)	0.85
WHO-QOL physical dimension	0.4 (-2.9, 3.7)	0.81	1.4 (-1.5, 4.3)	0.36	1.0 (-1.6, 3.5)	0.48
WHO-QOL mental dimension	1.3 (-2.5, 5.1)	0.54	1.4 (-2.1, 4.9)	0.48	1.3 (-1.8, 4.4)	0.44
IES-2 total	0.3 (-0.3, 0.9)	0.42	-0.0 (-0.6, 0.6)	0.98	0.4 (-0.1, 0.8)	0.13

Data shown as mean differences with corresponding 95% confidence intervals and p-values based on analysis of covariance including the baseline outcome value, baseline energy intake, and baseline physical activity as covariates.

BMD=bone mass density, BMI=body mass index, BW=body weight, DBP=diastolic blood pressure, EI=energy intake, FFM=fat-free mass, FM=fat mass, IES-2=Overall Intuitive Eating Scale-2 score (values in the interval 1-5), SBP=systolic blood pressure, VF=visceral fat, WC=waist circumference, WHO-QOL=WHO quality of life score (values in the interval 4-20).

Table S5. Pairwise comparisons of means at end of study (after 8 weeks) based on complete-case analyses adjusted for physical activity (n=39).

	**Mindful eating vs. control (n=7 vs n=10)**		**YogaDance vs. control (n=8 vs n=10)**		**Mindful eating+YogaDance vs. control (n=14 vs n=10)**	
	Estimate	p-value	Estimate	p-value	Estimate	p-value
**Outcome**						
FM, kg	-0.9 (-2.5, 0.8)	0.29	-0.1 (-1.6, 1.5)	0.95	-0.9 (-2.3, 0.5)	0.19
FFM, kg	0.8 (-0.5, 2.0)	0.22	-0.1 (-1.3, 1.2)	0.92	0.6 (-0.5, 1.6)	0.29
BW, kg	-0.8 (-3.0, 1.4)	0.46	0.0 (-2.1, 2.2)	0.82	-0.2 (-2.0, 1.7)	0.90
Fat percent, %	-0.5 (-1.7, 0.7)	0.37	-0.1 (-1.3, 1.0)	0.78	-1.1 (-2.1, -0.1)	**0.03**
BMI, kg/m^2	-0.2 (-1.0, 0.5)	0.56	0.1 (-0.6, 0.9)	0.67	0.00 (-0.6, 0.6)	0.99
WC, cm	-3.7 (-8.0, 0.5)	0.08	-0.9 (-5.2, 3.4)	0.35	-3.4 (-6.9, 0.2)	0.06
VF, g	-78 (-211, 56)	0.25	63 (-71, 196)	0.95	-36 (-146, 73)	0.50
BMD, g/cm^2	-0.01 (-0.03, 0,02)	0.68	0.00 (-0.03, 0.03)	0.39	0.00 (-0.02, 0.03)	0.89
SBP, mmHg	-3.2 (-13.0, 6.6)	0.51	-4.0 (-13.2, 5.2)	0.63	-0.6 (-8.8, 7.6)	0.88
DBP, mmHg	-0.7 (-6.7, 5.3)	0.81	1.4 (-4.4, 7.1)	0.18	2.0 (-3.0, 7.0)	0.41
WHOQOL physical dimension	0.9 (-4.7, 6.6)	0.27	1.0 (-0.7, 2.7)	0.24	1.0 (-0.4, 2.5)	0.14
WHOQOL mental dimension	1.1 (-0.4, 2.7)	0.15	0.4 (-1.2, 1.9)	0.61	1.3 (0.02, 2.6)	**0.047**
IES-2 total	0.5 (0.1, 0.9)	**0.01**	-0.01 (-0.4, 0.4)	0.97	0.4 (0.1, 0,8)	**0.01**
EI, kcal/day	-1568 (-4234, 1097)	0.24	39 (-2481, 2560)	0.97	1688 (-480, 3857)	0.12

Data shown as mean differences with corresponding 95% confidence intervals and p-values based on analysis of covariance including the baseline outcome value and baseline physical activity as covariates.

BMD=bone mass density, BMI=body mass index, BW=body weight, DBP=diastolic blood pressure, EI=energy intake, FFM=fat-free mass, FM=fat mass, IES-2=Overall Intuitive Eating Scale-2 score (values in the interval 1-5), SBP=systolic blood pressure, VF=visceral fat, WC=waist circumference, WHO-QOL=WHO quality of life score (values in the interval 4-20).

Table S6. Pairwise comparisons of means at end of study (after 8 weeks) based on complete-case analyses adjusted for both energy intake and physical activity (n=39).

	**Mindful eating vs. control (n=7 vs n=10)**		**YogaDance vs. control (n=8 vs n=10)**		**Mindful eating+YogaDance vs. control (n=14 vs n=10)**	
	Estimate	p-value	Estimate	p-value	Estimate	p-value
**Outcome**						
FM, kg	-1.1 (-2.8, 0.5)	0.17	-0.3 (-1.9, 1.3)	0.69	-0.8 (-2.1, 0.5)	0.24
FFM, kg	0.8 (-0.5, 2.1)	0.21	-0.0 (-1.3, 1.3)	0.97	0.6 (-0.5, 1.6)	0.30
BW, kg	-1.0 (-3.2, 1.4)	0.40	-0.2 (-2.4, 2.1)	0.89	-0.1 (-2.0, 1.8)	0.92
Fat percent, %	-0.8 (-1.9, 0.4)	0.18	-0.3 (-1.3, 0.8)	0.63	-0.96 (-1.89, -0.02)	**0.045**
BMI, kg/m^2	-0.3 (-1.1, 0.5)	0.47	0.0 (-0.7, 0.8)	0.90	0.00 (-0.6, 0.7)	0.96
WC, cm	-4.6 (-8.9, -0.3)	**0.04**	-1.7 (-6.0, 2.6)	0.43	-3.0 (-6.5, 0.4)	0.08
VF, g	-98 (-231, 35)	0.14	45 (-88, 177)	0.50	-23 (-131, 86)	0.67
BMD, g/cm^2	-0.01 (-0.04, 0,03)	0.76	0.00 (-0.03, 0.03)	0.92	0.00 (-0.03, 0.03)	0.92
SBP, mmHg	-3.8 (-14.1, 6.5)	0.45	-4.2 (-13.7, 5.2)	0.37	-0.5 (-8.8, 7.8)	0.90
DBP, mmHg	-0.5 (-6.8, 5.8)	0.88	1.5 (-4.4, 7.3)	0.62	1.9 (-3.2, 7.0)	0.45
WHOQOL physical dimension	1.0 (-0.8, 2.8)	0.27	1.0 (-0.7, 2.8)	0.25	1.0 (-0.4, 2.5)	0.16
WHOQOL mental dimension	1.0 (-0.6, 2.5)	0.23	0.3 (-1.3, 1.9)	0.70	1.4 (0.1, 2.7)	**0.04**
IES-2 total	0.5 (0.1, 0.9)	**0.02**	-0.00 (-0.4, 0.4)	>0.99	0.5 (0.2, 0,8)	**0.01**

Data shown as mean differences with corresponding 95% confidence intervals and p-values based on analysis of covariance including the baseline outcome value, baseline energy intake, and baseline physical activity as covariates.

BMD=bone mass density, BMI=body mass index, BW=body weight, DBP=diastolic blood pressure, FFM=fat-free mass, FM=fat mass, IES-2=Overall Intuitive Eating Scale-2 score (values in the interval 1-5), SBP=systolic blood pressure, VF=visceral fat, WC=waist circumference, WHO-QOL=WHO quality of life score (values in the interval 4-20).
